# Lake Erie ice is a repository of organisms

**DOI:** 10.1128/mra.01094-23

**Published:** 2024-02-27

**Authors:** Opeoluwa F. Iwaloye, Brenna Michaud, Tessa Alloy, Nigel D'Souza, R. Michael L. McKay, Paul Morris, Colby Gura, Scott O. Rogers

**Affiliations:** 1 Department of Biological Sciences, Bowling Green State University, Bowling Green, Ohio, USA; Montana State University, Bozeman, USA

**Keywords:** metagenome, metatranscriptome, great lakes, cyanobacteria, anthropogenic

## Abstract

Organism abundance and diversity were assessed in Lake Erie ice samples using sequences derived from a combined metagenomic and metatranscriptomic analysis. The 68,417 unique sequences were from Bacteria (77.5%), Eukarya (22.3%), and Archaea (0.2%) and indicated diverse species of organisms from 32 bacterial, 8 eukaryotic, and 2 archaeal taxonomic groups.

## ANNOUNCEMENT

Lake ice entraps enormous numbers of microbes, many of which remain viable (1–8). As the ice melts, the organisms are released into the water, potentially adding to the biodiversity in the lakes. Dead organisms provide carbon sources for the living organisms in the lake water. While lake water has been studied extensively (9–20), few such studies have been conducted on lake ice. Here, we report details of DNA and RNA sequences from Lake Erie ice.

A full-thickness surface ice sample (30 × 30 × 45 cm) was collected from Station 84 (42.05 N, −81.55 W) in the central basin of Lake Erie in January 2010. A subsample (8 × 8 × 15 cm) was immersed in 5% sodium hypochlorite, then rinsed with 2.0 L of sterile reverse osmosis purified water (18 MΩ, <1 ppm total organic carbon) followed by melting at room temperature in a sterile funnel and collected in sterile 50 mL screwcap tubes that were immediately frozen at −20°C. A total of 250 mL was thawed and subjected to ultracentrifugation (100,000 × *g* for 16 h at 4°C) to concentrate cells and nucleic acids. Pellets were rehydrated in 50 µL of sterile 0.1× TE [1 mM Tris (pH 7.5), 0.1 mM EDTA, 4°C], details described previously (3, 7, 8).

**Fig 1:**
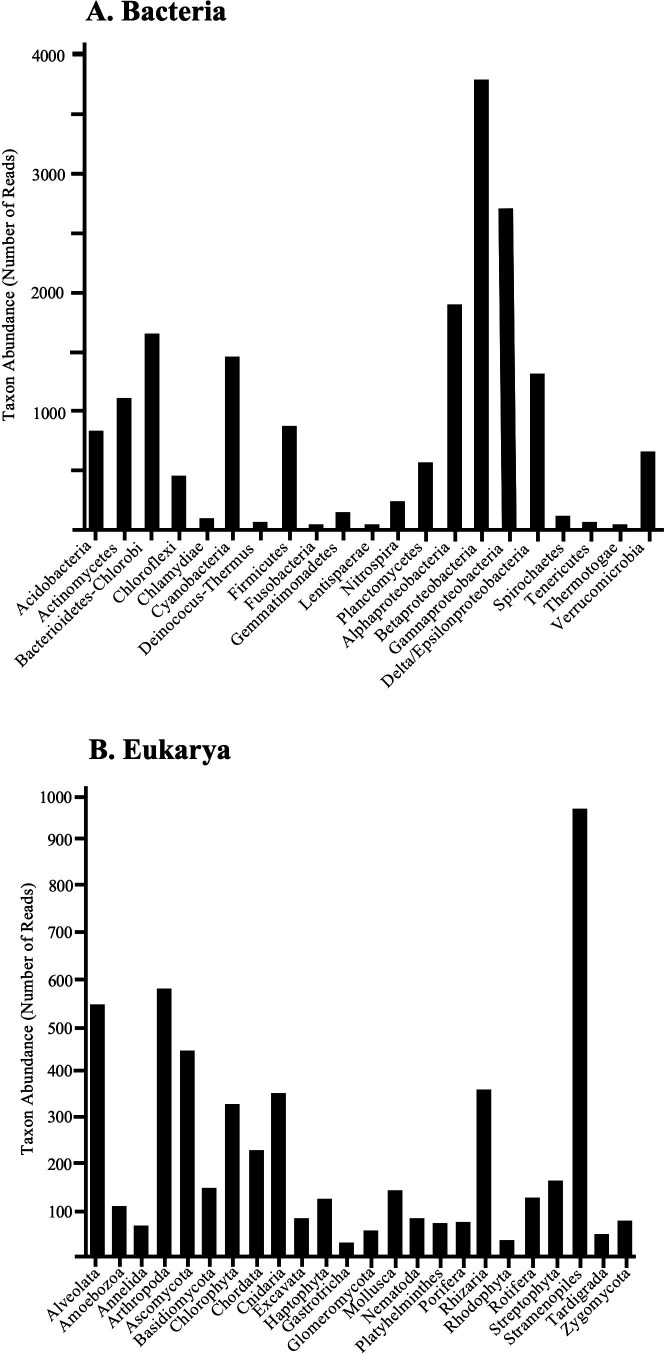
Abundance of Bacteria and Eukarya. (A) Total numbers of unique sequences (DNA and RNA) from each major taxon of Bacteria. Those with fewer than 20 sequences (not shown in the figure) were from Aquificae, Armatimonadetes, Caldisericia, Chlorochromatium, Chrysiogenetes, Cloacimonetes, Deferribacderes, Elusimicrobia, Fibrobacteres, Synergistota, Thermodesulfobacteriota, and Zetaproteobacteria.** (B)** Total numbers of unique sequences (DNA and RNA) from each major taxon of Eukarya. Those with fewer than 20 sequences (not shown in the figure) were from Acanthocephala, Blastocladiomycota, Brachiopoda, Bryozoa, Ctenophora, Echinodermata, Entorrhizomycota, Glaucophyta, Kinorhyncha, Nemertea, Placozoa, Phoronida, Pripulida, Spinuncula, and Xenacoelomorpha.

Nucleic acid extraction was performed using MinElute Spin Columns (QIAGEN, Valencia, CA, USA). The eluted nucleic acids were precipitated in ethanol, pelleted, dried, and rehydrated in 10 µL of 0.1× TE. DNA copies of the RNA were produced with a cDNA kit using random hexamer primers (Invitrogen SuperScript Choice System, Invitrogen, Grand Island, NY, USA). The resulting solution contained both DNA (metagenomic portion) and cDNA (metatranscriptomic portion). Adapters (AATTCGCGGCCGCGTCGAC, dsDNA) were ligated to each end of the cDNA and DNA using T4 DNA ligase. For pyrosequencing, 454-specific primers, one with 454 sequence A (underlined): CGTATCGCCTCCCTCGCGCCATCAGAATTCGCGGCCGCGTCGAC and the other with 454 sequence B (underlined): CTATGCGCCTTGCCAGCCCGCTCAGAATTCGCGGCCGCGTCGAC, were added to the nucleic acids using PCR (4 min at 94°C, followed by 40 cycles of 1 min at 94°C, 2 min at 55°C, 72°C, followed by 10 min at 72°C). The amplified nucleic acids were quantified on agarose gels and subjected to sequencing using a 454 GS Junior System (Roche Corporation, Indianapolis, IN, USA) by Roche staff, who also performed sequence filtering to extract high-quality reads (eliminating duplicates, reads < 20 nt, and reads > 20% ambiguous nucleotides). This reduced the reads from a total of 122,460 to 68,417 high-quality reads, with mean lengths of 390 nt. High-quality reads were subjected to MegaBLAST analysis, using a cut-off *e*-value of 10^−10^. Default parameters were used for all software. Taxonomic assignments were made based on the top BLAST matches. When the top BLAST match was taxonomically unknown, the next sequence within the top 10 that had a taxonomic designation was chosen. The number of sequences and the species abundances in each major taxonomic group were determined (Fig. 1; Table 1). Additional details were reported elsewhere (21).

**TABLE 1:** Total number of species by major taxon, based on top BLAST matches to DNA and RNA sequences

Domain	Major taxon	Number of species
Archaea	Euryarchaeota	19
	Thaumarchaeota	5
Bacteria	Acidobacteria	16
	Actinomycetes	228
	Armatimonadetes	2
	Aquificae	7
	Bacteroidetes	157
	Caldisericia	1
	Chlorobi	2
	Chlorochromatium	1
	Chloroflexi	16
	Chlamydia	14
	Chrysiogenetes	1
	Cloacimonetes	1
	Cyanobacteria	170
	Deferribacteres	3
	Deinococcus-Thermus	14
	Elusimicrobia	1
	Endomicrobia	1
	Fibrobacteres	1
	Firmicutes	270
	Fusobacter	9
	Gemmatimonadetes	1
	Lentisphaerae	2
	Nitrospira	6
	Planctomycetes	22
	Proteobacteria	
	Alphaproteobacteria	282
	Betaproteobacteria	270
	Deltaproteobacteria	98
	Gammaproteobacteria	427
	Zetaproteobacteria	1
	Solibacteres	3
	Spirochaetia	25
	Synergistia	5
	Tenericutes	24
	Thermodesulfobacteria	3
	Thermotogae	18
	Verrucomicrobia	18
Eukarya	Amoebozoa	19
	Apusozoa	5
	Archaeplastida	
	Anthrocerotophyta	1
	Bryophyta	8
	Chlorophyta	90
	Gnetophyta	1
	Lycophyta	1
	Marchantiophyta	2
	Polypodiophyta	2
	Rhodophyta	10
	Streptophyta	43
	Excavata	28
	Hacrobia	
	Centroheliozoa	1
	Cryptophyta	9
	Opisthokonta	
	Fungi	
	Ascomycota	72
	Basidiomycota	37
	Blastoclatiomycetes	1
	Chytridiomycota	10
	Crisidiscoidea	1
	Glomeromycota	4
	Kickxellomycota	1
	Microsporidia	1
	Mucoromycota	4
	Zygomycota	6
	Animalia	
	Annelida	12
	Arthropoda	87
	Brachiopoda	1
	Bryozoa	2
	Choanoflagellates	6
	Chordata	41
	Cnidaria	68
	Ctenophora	2
	Echinodermata	1
	Filasterea	1
	Gastrotrichia	3
	Kinorhyncha	1
	Mollusca	44
	Nematoda	16
	Nematomorpha	2
	Nemertea	3
	Phoronida	1
	Platyhelminthes	21
	Porifera	9
	Priapulida	2
	Rotifera	17
	Sipuncula	1
	Tardigrada	4
	Xenacoelomorpha	4
	SAR	
	Alveolata	34
	Rhizaria	35
	Stamenopiles	96

## Data Availability

The raw reads were deposited in the NCBI database PRJNA966190, SAMN34539107, SRR24412645.

## References

[1] D’Elia T, Veerapaneni R, Rogers SO. 2008. Isolation of microbes from Lake Vostok accretion ice. Appl Environ Microbiol 74:4962–4965. doi:10.1128/AEM.02501-07 18552196 PMC2519340

[2] D’Elia T, Veerapaneni R, Theraisnathan V, Rogers SO. 2009. Isolation of fungi from Lake Vostok accretion ice. Mycologia 101:751–763. doi:10.3852/08-184 19927741

[3] Gura C, Rogers SO. 2020. Metatranscriptomic and metagenomic analysis of biological diversity in subglacial Lake Vostok (Antarctica). Biology (Basel) 9:55. doi:10.3390/biology9030055 32188079 PMC7150893

[4] Knowlton C, Veerapaneni R, D’Elia T, Rogers SO. 2013. Analysis of ancient ice core sections from Greenland and Antarctica. Biology (Basel) 2:206–232. doi:10.3390/biology2010206 24832659 PMC4009855

[5] Ma L-J, Rogers SO, Catranis CM, Starmer WT. 2000. Detection and characterization of ancient fungi entrapped in glacial ice. Mycologia 92:286–295. doi:10.1080/00275514.2000.12061156

[6] Rivkina EM, Friedmann EI, McKay CP, Gilichinsky DA. 2000. Metabolic activity of permafrost bacteria below the freezing point. Appl Environ Microbiol 66:3230–3233. doi:10.1128/AEM.66.8.3230-3233.2000 10919774 PMC92138

[7] Rogers SO, Shtarkman YM, Koçer ZA, Edgar R, Veerapaneni R, D’Elia T. 2013. Ecology of subglacial Lake Vostok (Antarctica) based on metagenomic/metatranscriptomic analyses of accretion ice. Biology (Basel) 2:629–650. doi:10.3390/biology2020629 24832801 PMC3960894

[8] Shtarkman YM, Koçer ZA, Edgar R, Veerapaneni RS, D’Elia T, Morris PF, Rogers SO. 2013. Subglacial Lake Vostok (Antarctica) accretion ice contains a diverse set of sequences from aquatic, marine and sediment-inhabiting bacteria and eukarya. PLoS One 8:e67221. doi:10.1371/journal.pone.0067221 23843994 PMC3700977

[9] Baker DBD, Richards RPR. 2002. Phosphorus budgets and riverine phosphorus export in Northeastern Ohio watersheds. J Environ Qual 31:96–108. doi:10.2134/jeq2002.9600 11837450

[10] Beall BFN, Twiss MR, Smith DE, Oyserman BO, Rozmarynowycz MJ, Binding CE, Bourbonniere RA, Bullerjahn GS, Palmer ME, Reavie ED, Waters LMK, Woityra LWC, McKay RML. 2016. Ice cover extent drives phytoplankton and bacterial community structure in a large north-temperate lake: implications for a warming climate. Environ Microbiol 18:1704–1719. doi:10.1111/1462-2920.12819 25712272

[11] Beeton AM. 1965. Eutrophication of the St. Lawrence great lakes. Limnol Oceanog 10:240–254. doi:10.4319/lo.1965.10.2.0240

[12] Bertilsson S, Burgin A, Carey CC, Fey SB, Grossart H-P, Grubisic LM, Jones ID, Kirillin G, Lennon JT, Shade A, Smyth RL. 2013. The under-ice microbiome of seasonally frozen lakes. Limnol Oceanog 58:1998–2012. doi:10.4319/lo.2013.58.6.1998

[13] Bielewicz S, Bell E, Kong W, Friedberg I, Priscu JC, Morgan-Kiss RM. 2011. Protist diversity in a permanently ice-covered Antarctic Lake during the polar night transition. ISME J 5:1559–1564. doi:10.1038/ismej.2011.23 21390078 PMC3160681

[14] Butler TM, Wilhelm AC, Dwyer AC, Webb PN, Baldwin AL, Techtmann SM. 2019. Microbial community dynamics during lake ice freezing. Sci Rep 9:6231. doi:10.1038/s41598-019-42609-9 30996247 PMC6470161

[15] Kemp ALW, MacInnis GA, Harper NS. 1977. Sedimentation rates and a revised sediment budget for lake Erie. J Great Lakes Res 3:221–233. doi:10.1016/S0380-1330(77)72253-1

[16] Rankin D. 2002. Freshwater ecosystems and human populations: great lakes case study, p 61–84. In Krchnak KM, Markam VD, Thorne N, Coppock J (ed), Human populations and freshwater resrources: U.S. cases and international perspectives. Yale University Press, New Haven, Connecticut.

[17] Reinl KL, Harris TD, North RL, Almela P, Berger SA, Bizic M, Burnet SH, Grossart H-P, Ibelings BW, Jakobsson E, et al.. 2023. Blooms also like it cold. Limnol Oceanogr Letters 8:546–564. doi:10.1002/lol2.10316

[18] Rogozin DY, Zykov VV, Chernetsky MY, Degermendzhy AG, Gulati RD. 2009. Effect of winter conditions on distributions of anoxic phototrophic bacteria in two meromictic lakes in Siberia, Russia. Aquat Ecol 43:661–672. doi:10.1007/s10452-009-9270-7

[19] Twiss MR, McKay RML, Bourbonniere RA, Bullerjahn GS, Carrick HJ, Smith REH, Winter JG, D’souza NA, Furey PC, Lashaway AR, Saxton MA, Wilhelm SW. 2012. Diatoms abound in ice-covered Lake Erie: an investigation of offshore winter limnology in Lake Erie over the period 2007 to 2010. J Great Lakes Res 38:18–30. doi:10.1016/j.jglr.2011.12.008

[20] Waples JT, Eadie B, Klump JV, Squires M, Cotner J, McKinley G, The Laurentian great lakes. 2008. North American continental margins: a synthesis and planning workshop. U.S. carbon cycle science program. Edited by B Hales, W Cai, G Mitchell, CL Sabine, and O Schofield

[21] Iwaloye OF. 2021. Metagenomics and metatranscriptomics of Lake Erie ice Master of science thesis, Bowling Green State University, Department of Biologicel Sciences., Bowling Green, OH

